# Hypochlorite sensing and real-time imaging with XY-01: A red-emitting fluorescent turn-on probe for living cells and colorectal cancer organoids

**DOI:** 10.17305/bb.2025.13312

**Published:** 2025-12-17

**Authors:** Yichun Xu, Zhihua Chen, Jun Su, Yanting Ding, Jiajing Zhou, Jiawei Zhao, Zhiyuan He, Yi Gong, Zhai Cai, Lei Cui, Junsong Han

**Affiliations:** 1 Department of Pathology, Shanghai Tongji Hospital, Tongji Hospital Affiliated to Tongji University, Shanghai, China; 2 National Engineering Research Center for Biochip, Shanghai Biochip Limited Corporation, Shanghai, China; 3 College of Science, Shanghai University, Shanghai, China; 4 Department of General Surgery, Zhujiang Hospital, Southern Medical University, Guangzhou, China

**Keywords:** Fluorescent probe, hypochlorite, organoid, cell imaging, near-infrared emission

## Abstract

Hypochlorite (ClO^−^), a major reactive oxygen species generated in inflammation, is a potent biological oxidant involved in diverse physiological and pathological processes; therefore, sensitive detection of ClO^−^ is important for understanding disease pathophysiology and supporting early diagnosis and prevention. Here, we aimed to develop a physiologically compatible fluorescent tool for specific ClO^−^ sensing and imaging. We designed and synthesized a novel A–D–A type molecular fluorescent probe, XY-01, and characterized it by NMR, HRMS, UV–Vis and fluorescence spectroscopy. XY-01 operates through ClO^−^-triggered oxidation of a thioformyl group (C=S) to a carbonyl (C=O), which restores intramolecular charge transfer and produces a prominent fluorescence turn-on signal. In PBS (pH 7.4), XY-01 responded to ClO^−^ within 1 min with strong red emission at 666 nm and a large Stokes shift (∼167 nm), showed high selectivity against common ions and reactive species, and achieved a detection limit of 3.39 µM within the biologically relevant range. Cytotoxicity assays indicated negligible toxicity, enabling real-time confocal imaging of ClO^−^ distribution in HCT-116 cells and colorectal cancer organoids. Collectively, XY-01 is a simple, sensitive, and low-toxicity probe that provides a promising platform for optical sensing and imaging of hypochlorite in living cells and organoids.

## Introduction

Inflammation within biological systems is intricately linked to the levels of reactive oxygen species (ROS) [1, 2]. Hypochlorite (ClO^−^) is a significant ROS that plays a multifaceted role in various physiological and pathological processes [3]. It is produced via the catalysis of chloride ions and hydrogen peroxide (H_2_O_2_) by myeloperoxidase (MPO) [4, 5]. Excessive ClO^−^ can oxidize critical biomolecules, including proteins, nucleic acids, lipids, and enzymes, resulting in substantial tissue damage, inflammation, and the onset of various diseases [6–10]. Elevated levels of ClO^−^ exacerbate oxidative stress, which can compromise cell membranes, impair intercellular adhesion, and alter cellular viscosity [11]. These molecular changes further amplify the immune response and disrupt normal cellular functions, contributing to the progression of pathological conditions. Consequently, robust detection of ClO^−^ in living organisms is essential for elucidating its roles in disease pathophysiology [12, 13].

**Scheme 1.**
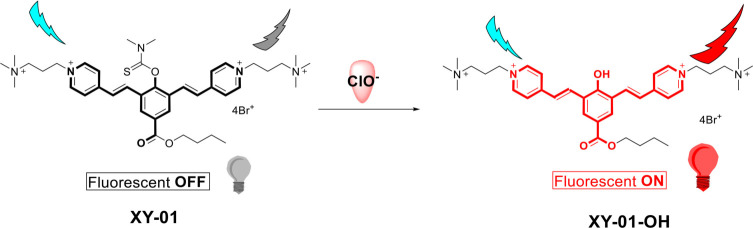
**Proposed reaction mechanism of probe XY-01 with ClO^−^.** The interaction of XY-01 with ClO^−^ results in the formation of the oxidized product XY-01-OH, thereby transitioning the probe from a fluorescence OFF state to a fluorescence ON state.

Numerous methods exist for detecting hypochlorite, including iodometric titration, colorimetry, chemiluminescence, coulometry, and radiolysis [14–16]. However, these techniques are often complex and may not be compatible with physiological environments. Due to their high selectivity, rapid response times, and low toxicity, fluorescrent probes have been developed for a variety of substrates. Fluorophores are critical in several scientific and technological applications, including bioimaging, diagnostics, and materials science [17–27]. While existing fluorescent probes demonstrate excellent performance in terms of photostability, response time, sensitivity, and selectivity, many are poorly water-soluble and unsuitable for use in living cells [28]. Moreover, the majority of available fluorescent probes require organic solvents, which are incompatible with aqueous physiological environments [29, 30]. This limitation highlights the necessity for detection methods that exhibit enhanced sensitivity and selectivity, specifically those that can operate effectively in aqueous conditions and the complex physiological milieu of living cells. Detecting ClO^−^ within complex biological structures, such as living organoids, poses additional challenges due to the intricate interactions and diffusion properties inherent to these three-dimensional models. Despite these obstacles, the ongoing development of advanced fluorescent probes offers promising potential for improving our capacity to monitor and comprehend ClO^−^-related processes in living organisms.

In this study, we synthesized a rapid hypochlorite-responsive acceptor–donor–acceptor (A–D–A) type fluorescent probe (Scheme 1). The molecular structure of probe XY-01 was characterized using ^1^H nuclear magnetic resonance (NMR), ^13^C NMR, high-resolution mass spectrometry (HRMS), ultraviolet–visible (UV–Vis), and fluorescence spectroscopy. The probe exhibited a substantial Stokes shift (approximately 167 nm) and responded to ClO^−^ within one minute, with enhanced fluorescence intensity at 666 nm. The novel fluorescent probe XY-01 is suitable for optical sensing and imaging of hypochlorite within living cells and organoids, characterized by straightforward operation, low toxicity, high sensitivity, and high selectivity. Furthermore, the fluorescent probe XY-01 demonstrated excellent adaptability to physiological conditions.

## Materials and methods

### Preparation of the fluorescent probe

Chemical reagents and solvents utilized in this study were obtained from commercial suppliers and used directly without additional purification. ^1^H NMR and ^13^C NMR spectra were recorded on a Bruker AV 500 spectrometer at room temperature. UV–Vis absorption spectra were acquired using a Techcomp UV2310II spectrophotometer, while photoluminescence (PL) spectra were measured on a Hitachi F-4500. Fluorescence images of cells and organoids were captured using confocal microscopy (Leica Stellaris5).

**Scheme 2.**
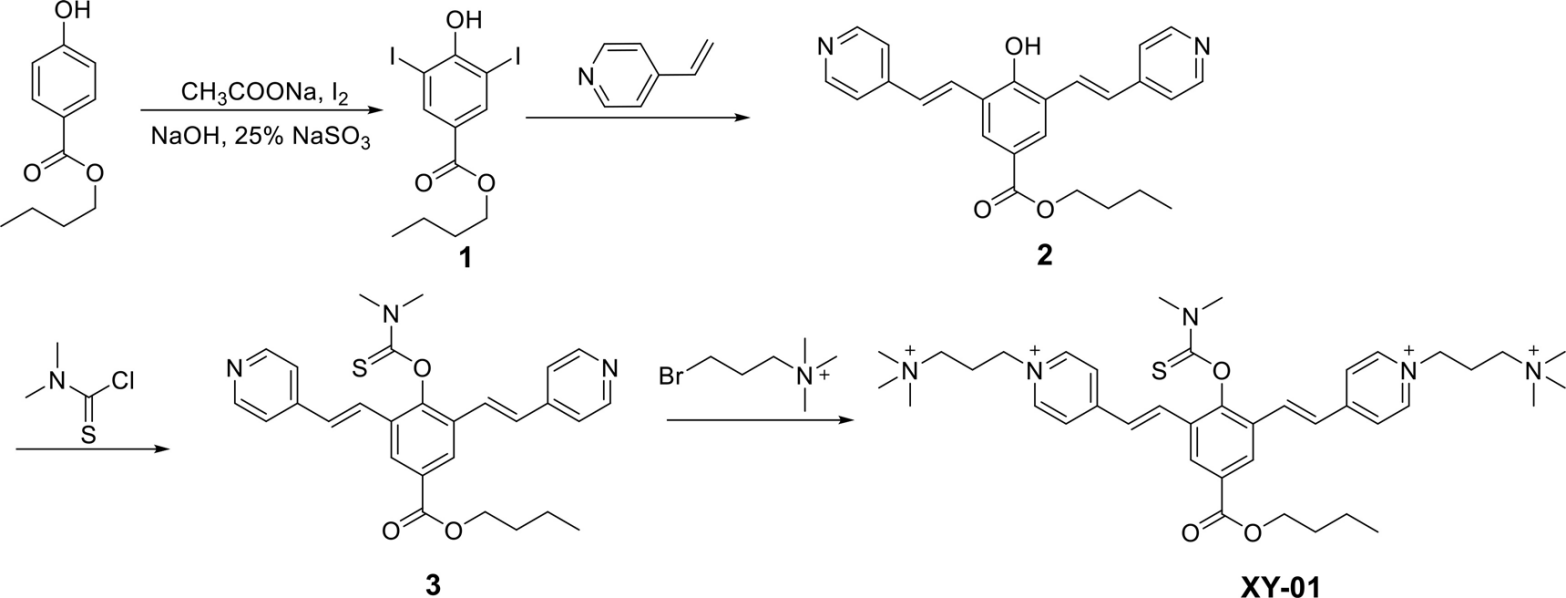
**Synthesis route of probe XY-01.** The synthesis of XY-01 is illustrated through intermediates 1–3, which were produced following established protocols in the literature [31, 32]. The structure of the final probe, XY-01, was validated using ^1^H NMR, ^13^C NMR, and high-resolution mass spectrometry (HRMS).

**Scheme 3.**
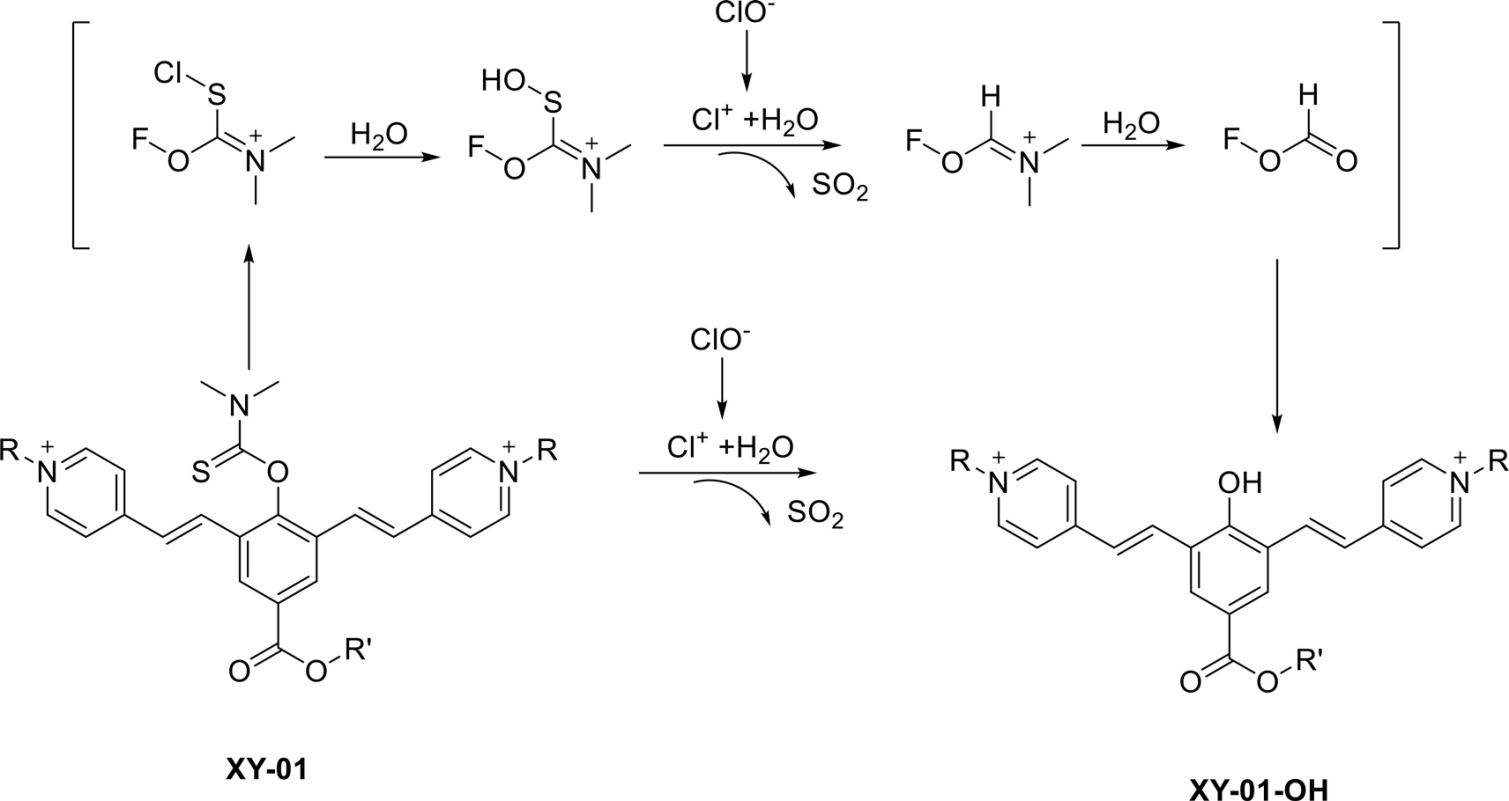
**Reaction mechanism of probe XY-01.** The chloride ion (ClO^−^) oxidizes the thioformyl group in XY-01, yielding the corresponding carbonyl product (XY-01-OH). This reaction restores intramolecular charge transfer (ICT) and generates a fluorescence turn-on response, accompanied by an approximately 167 nm redshift in both absorption and emission.

The proposed mechanism for the designed probe is illustrated in Scheme 1, while the synthetic route for probe XY-01 is depicted in Scheme 2. Compounds 1, 2, and 3 were synthesized according to the literature [31, 32]. Structural identification of XY-01 was confirmed through ^1^H and ^13^C NMR and HRMS spectrometry. The probe was dissolved in dimethyl sulfoxide (DMSO) to prepare a 10 mmol/L stock solution for subsequent use. XY-01 features a thioformyl group (–C=S) that serves as both the reactive moiety and the core unit disrupting ground-state intramolecular charge transfer (ICT). ClO^−^ selectively oxidizes this group to a carbonyl group (–C=O), enhancing the electron-withdrawing capacity of the product and reconstructing the ICT pathway by reducing the electron transfer energy barrier between donor (D) and acceptor (A) units, thus restoring ICT. We explicitly correlated this ICT recovery with spectral changes: the restored ICT induced a 167 nm redshift in the absorption/emission spectra and eliminated thioformyl-induced fluorescence quenching, resulting in significant fluorescence enhancement (turn-on signal) (Scheme 3).

### Cell culture

HCT-116 cells were procured from the National Collection of Authenticated Cell Cultures (Shanghai, China). These cells were cultured in RPMI-1640 medium supplemented with 10% (v/v) fetal bovine serum (FBS). The cells were maintained in a humidified atmosphere containing 5% CO_2_ and 95% air at 37 ^∘^C.

### Analysis of probe toxicity on HCT-116 cells

HCT-116 cells were seeded at a density of 1.0×10^5^ cells/well in a 96-well plate and cultured overnight. Cells were treated with the probe at various concentrations (0, 10, 50, 100, 200, and 400 µM) for one hour. Following treatment, cell viability was assessed using a CCK-8 assay in accordance with the manufacturer’s instructions.

### Confocal microscopy imaging of HCT-116 cells

To further evaluate the application of probe XY-01 for imaging in cancer cells, HCT-116 cells were again selected for cellular fluorescence imaging. Cells were seeded at a density of 1.0×10^5^ cells/well in a 96-well plate and cultured overnight. Commercial 7.5% NaOCl/HOCl was diluted and titrated with standardized sodium thiosulfate using a starch indicator, adjusted to pH 7.4 with 10 mM phosphate-buffered saline (PBS), and used within 24 h after dark storage at 4 ^∘^C to prevent degradation. Four experimental groups were established: the control group, the ClO^−^ addition group, the agonist group, and the antagonist group. In the control group, only 10 µM probe XY-01 was added to co-culture for 30 min. In the ClO^−^ addition group, cells were treated with 1 mM NaOCl/HOCl for 30 min and then washed. Subsequently, 10 µM probe XY-01 was added for co-culture for an additional 30 min. In the agonist group, HCT-116 cells were initially incubated with 1.0 µg/mL lipopolysaccharide (LPS) for 12 h, followed by incubation with 1.0 µg/mL phorbol 12-myristate 13-acetate (PMA) for one hour. Then, 10 µM probe XY-01 was introduced for 30 min. In the antagonist group, HCT-116 cells were first incubated with 200 µM 4-aminobenzoic acid hydrazide (ABAH) for three hours, followed by incubation with 1.0 µg/mL LPS for 12 h. The cells were then treated with 1.0 µg/mL PMA for one hour, followed by incubation with 10 µM probe XY-01 for 30 min. Finally, fluorescence imaging for all four groups was performed using a confocal microscope (Leica Stellaris5).

### Generation and identification of patient-derived organoids (PDOs)

Colorectal tumor tissues were acquired from patients undergoing surgical resection at Zhujiang Hospital of Southern Medical University, with written informed consent obtained. Ethical approval was granted by the institutional ethics committee (Approval No. 2023-KY-165-01). Three PDO lines were established from the surgically resected colorectal tumors according to established protocols [33]. These PDOs were characterized through immunofluorescence staining of colorectal cancer markers, including caudal type homeobox 2 (CDX2), cytokeratin 20 (CK20), pan-cytokeratin (PAN-CK), and Ki-67, to confirm their epithelial origin and cancer-specific characteristics [34, 35].

### Analysis of probe toxicity on colorectal cancer organoids

Three lines of colorectal cancer organoids were utilized to evaluate probe toxicity. The organoids were initially digested into single cells and collected. A single-cell suspension was then dispensed into a 96-well plate, with approximately 3000 cells per well. After 10 min of solidification in an incubator at 37 ^∘^C, 50 µL of medium was added to each well. After 48 h, the medium containing probe XY-01 at concentrations of 0, 10, 50, 100, 200, and 400 µM was replaced. Following 2 h of treatment with probe XY-01, ATP levels were assessed using CellTiter-Glo 3D (G9683, Promega).

### Confocal microscope imaging of colorectal cancer organoids

To further evaluate the application of probe XY-01 in imaging PDOs, colorectal cancer organoids were selected for cellular fluorescence imaging. The organoids were first digested into single cells and subsequently collected. The single-cell suspension was then dispensed into a 96-well plate, with approximately 3000 cells per well. After 10 min of solidification at 37 ^∘^C, 50 µL of medium was added to each well. The preparation of a 1 mM NaOCl/HOCl stock followed established methods. Four experimental groups were included: the control group, the ClO^−^ addition group, the agonist group, and the antagonist group. In the control group, only 10 µM probe XY-01 was added to co-culture with the organoids for 1 h. In the ClO^−^ addition group, the organoids were treated with 1 mM ClO^−^ (7.5% sodium hypochlorite aqueous solution) for 30 min, followed by washing. Subsequently, 10 µM probe XY-01 was added for another hour of co-culture. In the agonist group, the organoids were initially incubated with 1.0 µg/mL LPS for 12 h, followed by 1.0 µg/mL PMA for 1 hour. Then, 10 µM probe XY-01 was added and co-incubated with the organoids for 30 min. In the antagonist group, the organoids were incubated with 200 µM ABAH for 3 h, followed by 1.0 µg/mL LPS for 12 h, and then with 1.0 µg/mL PMA for 1 h, before final incubation with 10 µM probe XY-01 for 1 h. Fluorescence imaging experiments for all four groups were conducted using a confocal microscope (Leica Stellaris 5).

### Statistical analysis

Experimental values are expressed as mean ± SD. Statistical analyses were performed using GraphPad Prism 5.0 software to assess the significance of differences between groups, with thresholds set at *P < 0.05;* *P < 0.01; ***P < 0.001. All data represent the mean of triplicate experiments.

## Results

### Preparation of the fluorescence probe

The fluorescence probe XY-01 was synthesized as depicted in Scheme 2. Structural identification of the probe was confirmed by 1H, 13C NMR, and HRMS spectrometry (Figures S1–S4). High-performance liquid chromatography (HPLC) analysis of XY-01 oxidation by ClO^−^ revealed characteristic peaks for XY-01 (1.7 min) and its oxidized product XY-01-OH (6.2 min), with the latter peak gradually increasing upon ClO^−^ addition, supporting the mechanism by which XY-01 reacts with ClO^−^ to form XY-01-OH (Figure S5).

### Photophysical properties

**Figure 1.**
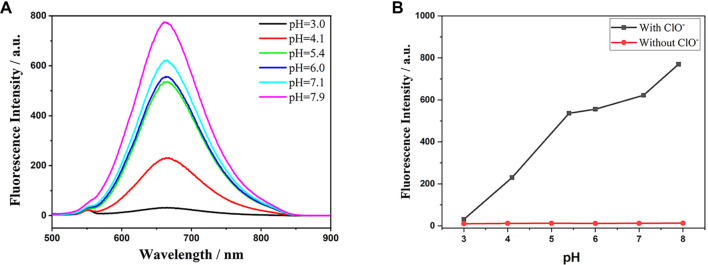
**pH-dependent fluorescence response of XY-01 toward hypochlorite in 10 mM PBS.** (A) Fluorescence emission spectra of XY-01 (10.0 µM) in the presence of ClO^−^ (1.0 mM) recorded at different pH values (3.0–7.9). (B) Fluorescence intensity at 666 nm (I_6__6__6_) of XY-01 alone and XY-01 + ClO^−^ as a function of pH (3.0–8.0), showing a gradual increase in signal at higher pH attributed to deprotonation of the phenolic hydroxyl group of XY-01-OH. Abbreviations: PBS: Phosphate-buffered saline; ClO^−^: Hypochlorite; I_6__6__6_: Fluorescence intensity at 666 nm.

To investigate the effect of pH on the fluorescence response of the probe, fluorescence intensities of XY-01 and the XY-01 + ClO^−^ system were measured in 10 mM PBS across a pH range of 3.0–8.0, as shown in Figure 1. As pH increased, the fluorescence intensity (I_6__6__6_) of the system gradually rose, attributed to the deprotonation of the phenolic hydroxyl group of XY-01-OH under alkaline conditions. Consequently, a buffer solution with pH 7.4 was selected for subsequent experiments.

**Figure 2.**
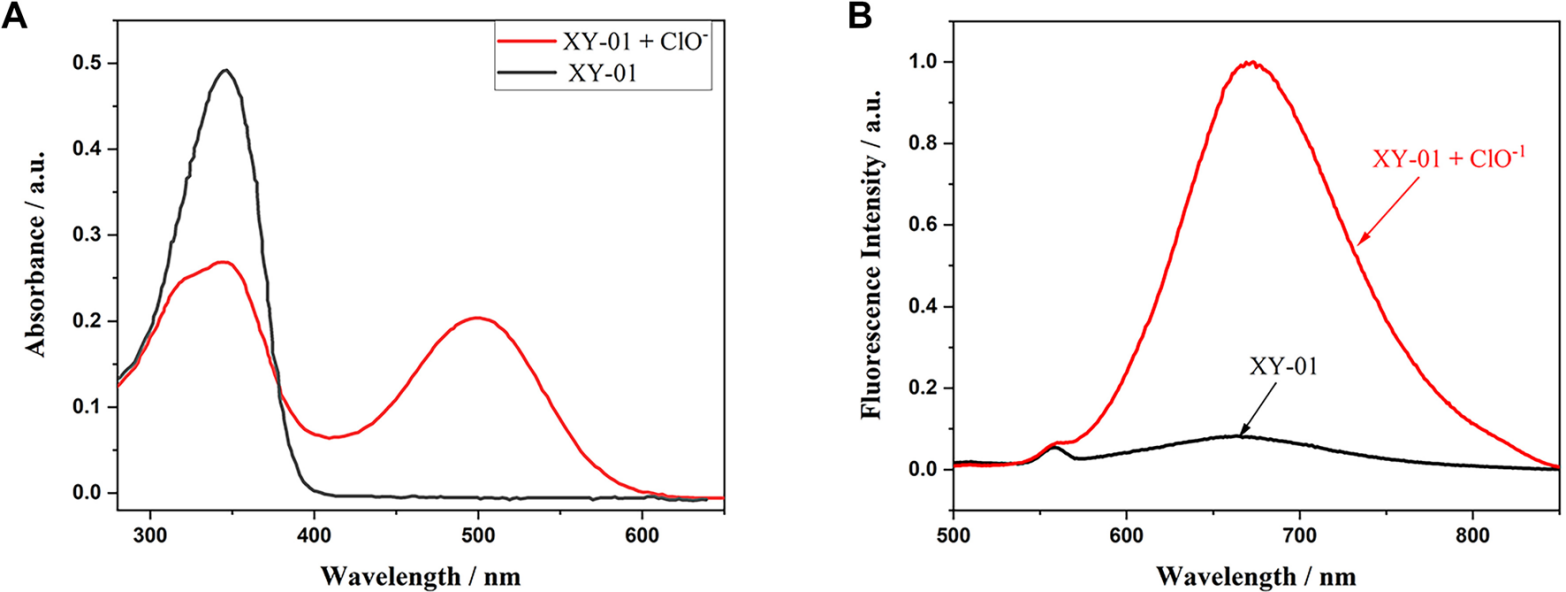
**UV–Vis and fluorescence response of XY-01 to hypochlorite in PBS (pH 7.4, 1×).** (A) Absorption spectra of XY-01 (10.0 µM) before (black) and after addition of ClO^−^ (1.0 mM, red), showing attenuation of the 342 nm band and appearance of a new absorption band at 499 nm (consistent with ICT restoration). (B) Fluorescence emission spectra of XY-01 (10.0 µM) without (black) and with ClO^−^ (1.0 mM, red) recorded at λ_ex_ ═ 499 nm (slit 5/5 nm), giving a strong turn-on emission at ∼666 nm (Stokes shift ∼167 nm). Abbreviations: PBS: Phosphate-buffered saline; ClO^−^: Hypochlorite; UV–Vis: Ultraviolet–visible; ICT: Intramolecular charge transfer.

The absorption spectra of XY-01 are shown in Figure 2, exhibiting a peak at 342 nm. As indicated in Figure 2A, XY-01 displayed a red fluorescence emission peak at approximately 666 nm. The UV–visible absorption and fluorescence properties of probe XY-01 in the presence of hypochlorite were investigated in PBS buffer (pH = 7.4, 1X). Upon ClO^−^ addition, the absorption at 342 nm decreased, while a new absorption peak emerged at 499 nm, intensifying with increasing ClO^−^ concentration, attributed to the restoration of the ICT mechanism. The thioformyl group (–C=S) of XY-01 serves as both the reactive moiety and the core unit that disrupts ground-state ICT. As shown in Figure 2B, the reaction product of probe XY-01 with ClO^−^ exhibited an excitation wavelength of 499 nm and an emission wavelength of 666 nm. The Stokes shift of XY-01 was calculated as the difference between the absorption maximum of its oxidized form (499 nm, consistent with the excitation wavelength *λ*_ex_) and the emission maximum (666 nm), resulting in a value of 167 nm, which is relatively large among organic molecular probes. In PBS solution (pH = 7.4, 1X), the reaction product of probe XY-01 with hypochlorite existed in both deprotonated and protonated forms, with the emission at 666 nm corresponding to the deprotonated form.

### Selectivity experiment

**Figure 3.**
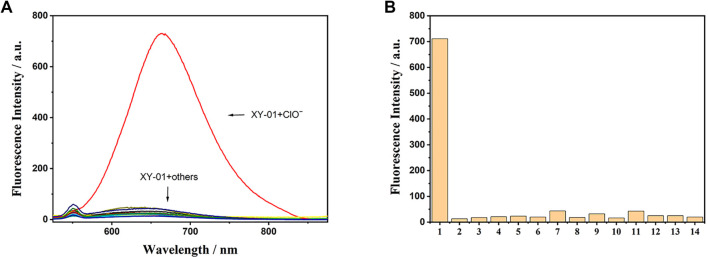
**Selectivity of probe XY-01 toward hypochlorite.** (A) Fluorescence emission spectra of XY-01 (10.0 µM) after incubation with ClO^−^ or other tested ions/ROS (each 1.0 mM) for 90 min at room temperature (λ_ex_ ═ 499 nm), showing a pronounced turn-on signal only in the presence of ClO^−^ (λ_em_ ≈ 666 nm). (B) Corresponding fluorescence intensities at 666 nm for XY-01 with different analytes (1–14): ClO^−^, S_2_O_3_^2−^, SO_4_^2−^, Br^−^, Cl^−^, Mg^2+^, SO_3_^2−^, HSO_3_^−^, Cu^2+^, CH_3_COO^−^, CO_3_^2−^, K^+^, OH^−^, H_2_O_2_. Abbreviations: ClO^−^: Hypochlorite; ROS: Reactive oxygen species; λ_ex_: Excitation wavelength; λ_em_: Emission wavelength.

Fluorescent probes are widely utilized to target molecules for cellular imaging. Given the complex intracellular environment, which contains a variety of ROS and ions, it is essential to assess the selectivity of probe XY-01. To evaluate the specific recognition ability of XY-01, a fluorescence spectroscopy experiment was conducted. Various ions were added to the XY-01 solution and incubated at room temperature for 90 min, followed by fluorescence spectral analysis. As illustrated in Figure 3, the fluorescence intensity at 666 nm exhibited no significant change with the addition of other ROS or ions. However, the introduction of ClO^−^ led to a substantial increase in fluorescence intensity. These results indicate that probe XY-01 demonstrates high selectivity and specificity for ClO^−^ in cellular environments. Additionally, upon the addition of ClO^−^, the solution’s color changed from colorless to red, visibly confirming that fluorescent probe XY-01 possesses strong anti-interference capabilities and reacts specifically with ClO^−^, even amid numerous interfering ions. A total of 13 different interfering analytes were tested, none of which significantly impacted the ability of probe XY-01 to detect ClO^−^.

### Response time

**Figure 4.**
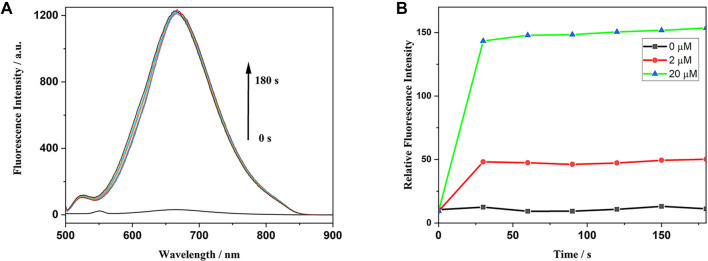
**Response kinetics of XY-01 toward hypochlorite.** (A) Time-resolved fluorescence emission spectra of XY-01 recorded from 0 to 180 s after addition of ClO^−^, showing a rapid signal increase with the emission maximum at 666 nm and equilibration within ∼1 min. (B) Time-dependent fluorescence intensity changes of XY-01 upon treatment with different ClO^−^ concentrations (0, 2, and 20 µM).

Response time is a critical factor in evaluating the performance of a probe and a key indicator of its adaptability to varying detection environments. To determine the optimal response time, we monitored changes in fluorescence intensity of the XY-01 + ClO^−^ system at 666 nm over time. The response time of probe XY-01 to hypochlorite was measured at different time intervals at room temperature. Figure 4A displays the fluorescence spectra of XY-01 at intervals ranging from 0 to 180 s following the addition of ClO^−^. Post-addition, the fluorescence intensity increased and reached equilibrium within one minute, peaking at a wavelength of 666 nm. The time-dependent fluorescence intensity changes of XY-01 upon the addition of ClO^−^ at varying concentrations (0, 2, and 20 µM) are illustrated in Figure 4B.

To further examine the probe’s sensitivity, concentration titration experiments were conducted. Utilizing the International Union of Pure and Applied Chemistry (IUPAC)-recommended formula for the limit of detection (LOD) ═ 3σ_blank/slope, the LOD of the probe was determined to be 3.39 µM, which is suitable for the physiological concentration range of hypochlorite (5–25 µM). Following the IUPAC-recommended formula for the limit of quantification (LOQ) ═ 10σ_blank/slope, the LOQ was established at 11.30 µM. Both the LOD and LOQ were determined using eight calibration points with three technical replicates for each concentration. These findings suggest that probe XY-01 can rapidly and sensitively detect ClO^−^. The fluorescence quantum yield (Φ) was assessed using a relative method, with Rhodamine 6G serving as the reference dye. Measurements were conducted in PBS solution (pH = 7.4, 1X) under identical excitation conditions (499 nm) for both the probe and the reference. The fluorescence quantum yields (Φ) for XY-01 and XY-01-OH were found to be 0.025 and 0.33, respectively.

### Cytotoxic effect of the probe on HCT-116 cells

**Figure 5.**
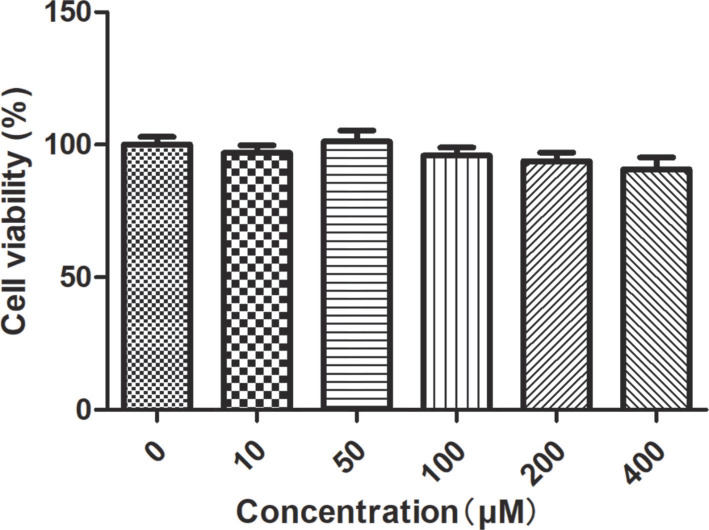
**Cytotoxicity evaluation of XY-01 in HCT-116 cells.** Cell viability after 1 h incubation with increasing concentrations of XY-01, determined by the CCK-8 assay; all treated groups maintained >95% viability relative to the untreated control. Abbreviations: CCK-8: Cell Counting Kit-8; HCT-116: Human colorectal carcinoma cell line.

The viability of HCT-116 cells post-treatment with the probe was evaluated using the CCK8 assay. The cytotoxicity of the probe against HCT-116 cells is illustrated in Figure 5. For cells treated for one hour, the viability across all treated groups remained above 95% compared to the control group. The CCK8 assay results indicated that the probe exhibited negligible cytotoxicity toward HCT-116 cells (Figure 5). We further assessed the probe’s cytotoxicity at 12 and 24 h; cell viability remained above 95% at concentrations ≤ 200 µM, underscoring its suitability for long-term live-cell imaging (Figure S6A and S6B).

**Figure 6.**
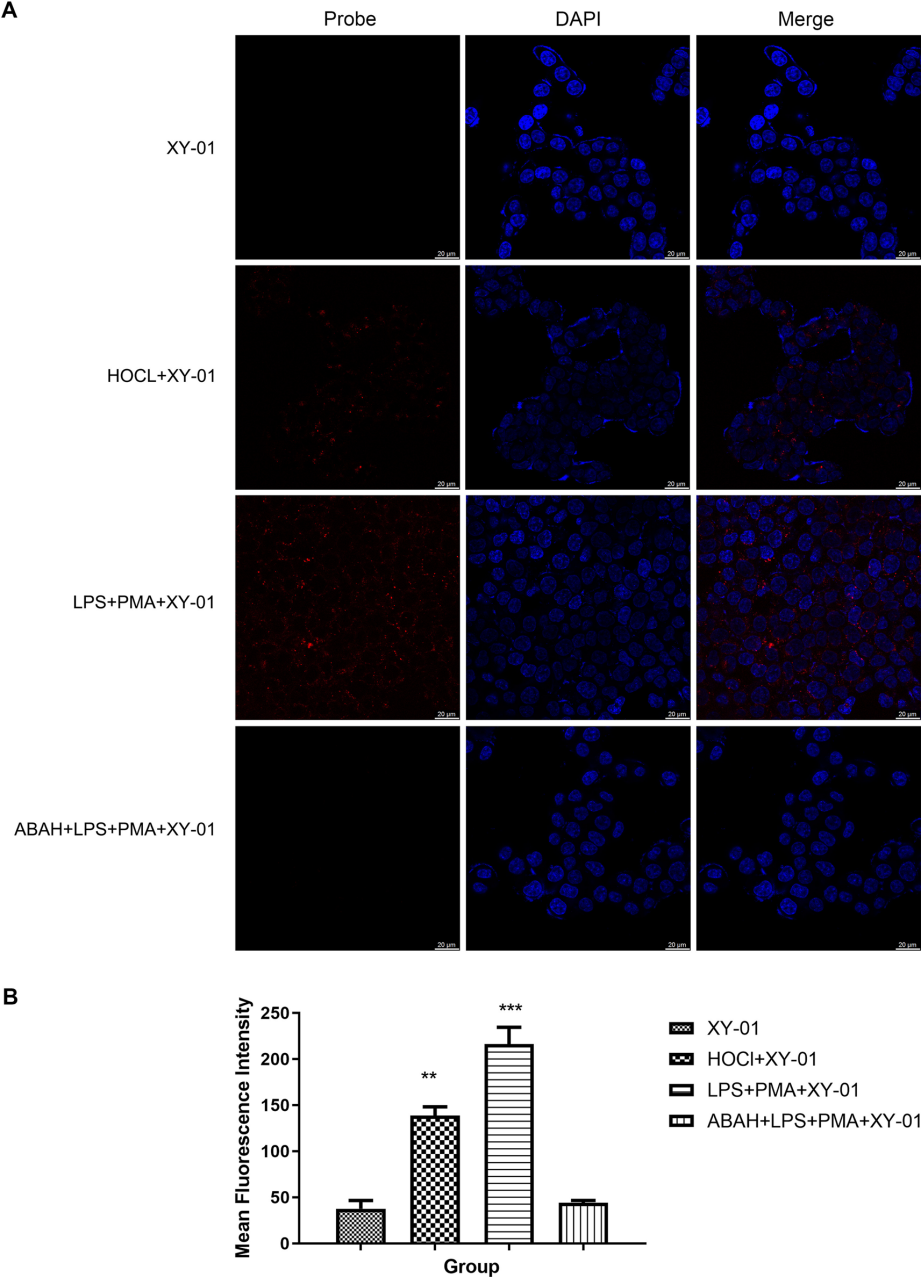
**Confocal fluorescence imaging of intracellular hypochlorite in HCT-116 cells using probe XY-01.** (A) Representative confocal images showing the XY-01 channel (Probe), nuclear staining (DAPI), and merged images for four conditions: cells incubated with XY-01 alone (10 µM, 30 min), cells pretreated with HOCl (1 mM, 30 min) followed by XY-01 staining, agonist-stimulated cells (LPS, 1.0 µg/mL, 12 h; then PMA, 1.0 µg/mL, 1 h) followed by XY-01 staining, and antagonist-treated cells (ABAH, 200 µM, 3 h) prior to LPS/PMA stimulation and XY-01 staining. Scale bar: 20 µm. Imaging settings: laser lines 405 nm/465 nm; detector HyD S1/HyD S2; pinhole 77.2 µm; gain 6.8/2.5; dwell time 1.575 µs. (B) Quantification of mean fluorescence intensity for each group (*P < 0.05, **P < 0.01, ***P < 0.001). Abbreviations: ABAH: 4-aminobenzoic acid hydrazide; DAPI: 4′,6-diamidino-2-phenylindole; HCT-116: Human colorectal carcinoma cell line; HOCl: Hypochlorous acid; HyD: Hybrid detector; LPS: Lipopolysaccharide; PMA: Phorbol 12-myristate 13-acetate.

**Figure 7.**
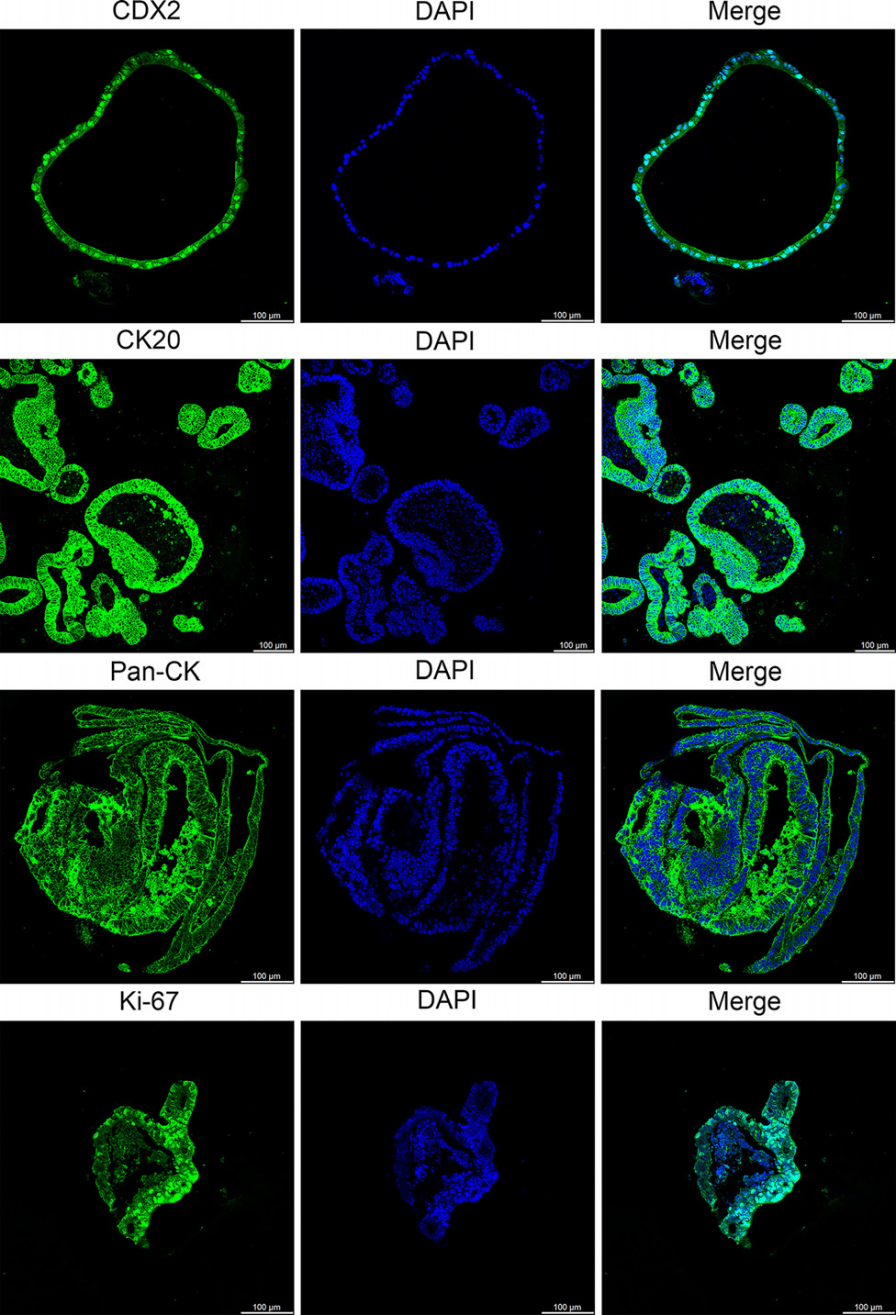
**Confocal microscopy images for the identification of colorectal cancer organoids.** Antibodies targeting CDX2 (intestinal epithelial cell marker), CK20 (intestinal epithelial cell marker), PAN-CK (epithelial cell marker), and Ki-67 (cell proliferation marker) were utilized. DAPI was employed to stain the nuclei. Scale bar: 100 µm; Laser wavelengths: 405 nm/488 nm; Detectors: HyD S1/HyD S2; Pinhole diameter: 53.1 µm; Gain settings: 21.4/2.5; Dwell time: 1.575 µs. Abbreviations: CDX2: Caudal type homeobox 2; CK20: Cytokeratin 20; PAN-CK: Pan-cytokeratin; Ki-67: Ki-67 proliferation-associated nuclear antigen; DAPI: 4′,6-diamidino-2-phenylindole; HyD: Hybrid detector.

### Fluorescence imaging of ClO^−^ in HCT-116 cells

Given the low cytotoxicity of probe XY-01, it was employed to detect intracellular ClO^−^ in HCT-116 cells. As presented in Figure 6A, HCT-116 cells incubated solely with probe XY-01 (10 µM) exhibited negligible fluorescence. Conversely, HCT-116 cells pretreated with ClO^−^ (1 mM) demonstrated a significant fluorescence enhancement (Figure 6B). Fluorescence was markedly increased in the agonist group (Figure 6C), while it remained scarcely detectable in the antagonist group (Figure 6D). These results affirm that probe XY-01 can effectively detect ClO^−^ in living cells.

### Cytotoxic effect of probe XY-01 on colorectal cancer organoids

PDOs are generated from epithelial cells and reflect the characteristics of corresponding tissues [36]. They serve as promising tools for drug screening and predicting patient responses [37]. To further evaluate the cytotoxic effect of probe XY-01, it was assessed in patient-derived colorectal cancer organoids, which recapitulate the heterogeneity and architecture of primary tumors. Three PDO lines were characterized through immunofluorescence staining for colorectal cancer markers, including CDX2, CK20, PAN-CK, and Ki-67. Results confirmed the epithelial origin and cancer-specific features of these PDO lines (Figure 7).

**Figure 8.**
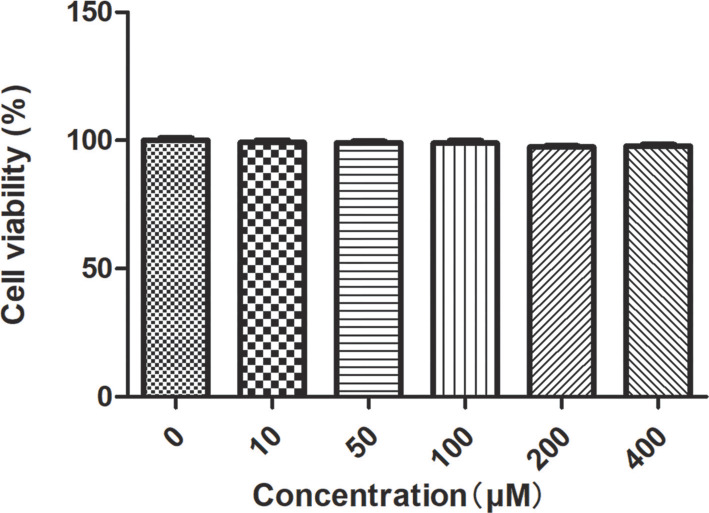
**Cytotoxicity assessment of XY-01 in colorectal cancer organoids.** Organoid viability after 2 h exposure to increasing concentrations of XY-01, measured by an ATP-based assay; all treated groups maintained >95% viability relative to the untreated control, indicating minimal cytotoxicity under the experimental conditions.

The cytotoxicity of the probe against the three colorectal cancer organoids is presented in Figure 8. For organoids treated for two hours, cell viability across all treated groups exceeded 95% compared to the control group. The ATP assay results demonstrated minimal cytotoxicity of the probe toward colorectal cancer organoids (Figure 8). We also evaluated the probe’s cytotoxicity at 12 and 24 h; cell viability remained above 95% at concentrations ≤ 400 µM, indicating its suitability for long-term live-organoid imaging (Figure S6C and S6D).

### Fluorescence imaging of ClO^−^ in colorectal cancer organoids

**Figure 9.**
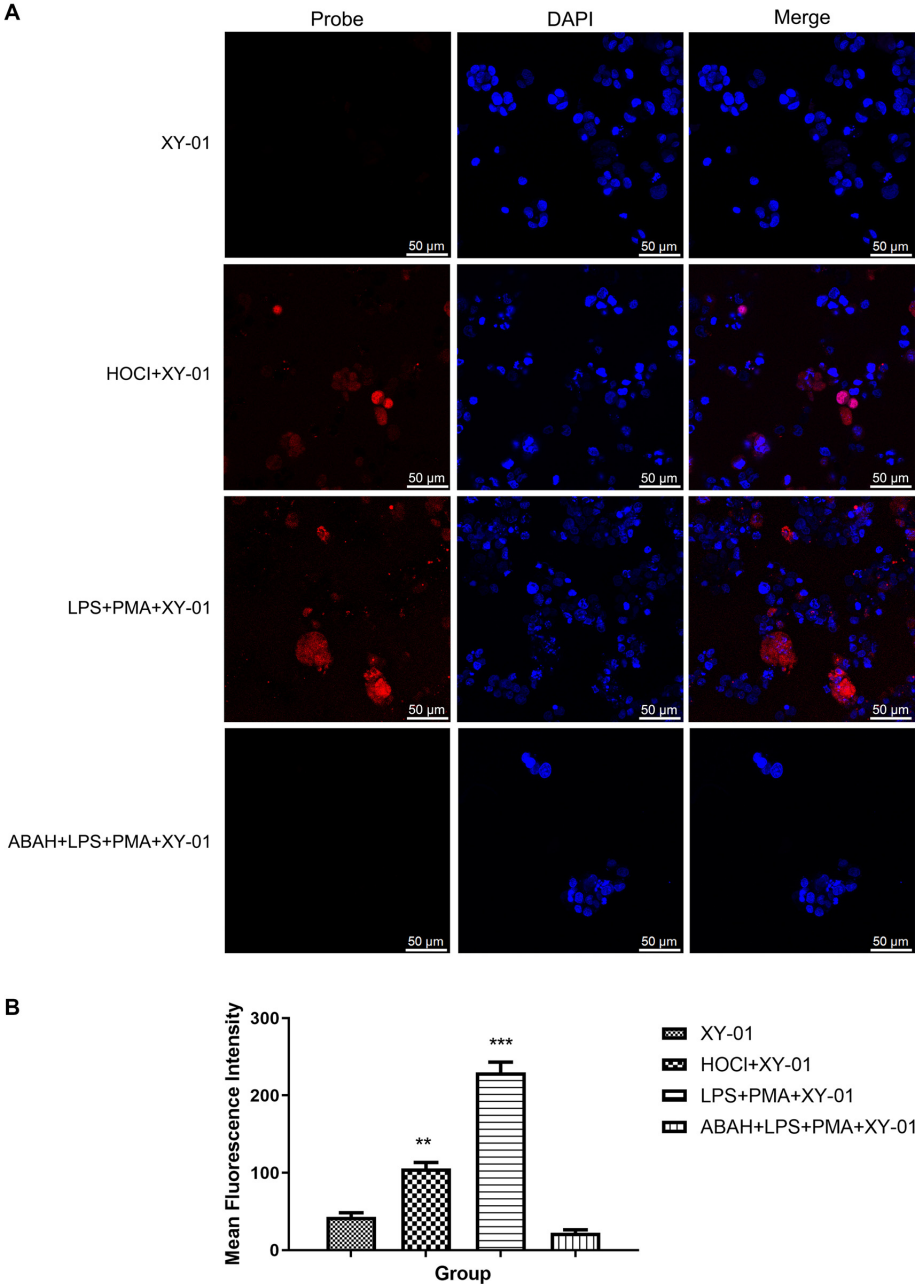
**Confocal fluorescence imaging of intracellular hypochlorite in colorectal cancer organoids using probe XY-01.** (A) Representative confocal images showing the XY-01 channel (Probe), nuclear staining (DAPI), and merged images under four conditions: organoids incubated with XY-01 alone (10 µM; negligible fluorescence), organoids pretreated with HOCl (1 mM) followed by XY-01 staining (enhanced fluorescence), agonist-stimulated organoids (LPS + PMA) followed by XY-01 staining (marked fluorescence increase), and antagonist-treated organoids (ABAH + LPS + PMA) followed by XY-01 staining (fluorescence strongly suppressed). Scale bar: 50 µm. Imaging settings: laser lines 405 nm/465 nm; detector HyD S1/HyD S2; pinhole 77.2 µm; gain 10.9/2.5; dwell time 1.575 µs. (B) Quantification of mean fluorescence intensity for each group (*P < 0.05, **P < 0.01, ***P < 0.001). Abbreviations: ABAH: 4-aminobenzoic acid hydrazide; DAPI: 4′,6-diamidino-2-phenylindole; HyD: Hybrid detector; LPS: Lipopolysaccharide; PMA: Phorbol 12-myristate 13-acetate.

Given the low cytotoxicity of probe XY-01, it was utilized to detect intracellular ClO^−^ in colorectal cancer organoids. As shown in Figure 9, colorectal cancer organoids incubated with probe XY-01 (10 µM) displayed negligible fluorescence. In contrast, organoids pretreated with ClO^−^ (1 mM) exhibited a remarkable fluorescence enhancement (Figure 9). Fluorescence in colorectal cancer organoids was significantly enhanced in the agonist group, while remaining scarcely detectable in the antagonist group. These findings demonstrate that probe XY-01 can effectively detect ClO^−^ in cancer organoids.

## Discussion

ClO^−^ mediates complex physiological and pathological functions, and its overproduction can lead to tissue damage, inflammation, and various diseases through the oxidation of key biomolecules [1, 38, 39]. While several fluorescent probes for ClO^−^ detection exhibit favorable photophysical properties, many are constrained by poor water solubility, limiting their application in living cells.

In this study, we synthesized a rapid hypochlorite-responsive A-D-A type fluorescent probe, XY-01, designed for the highly selective and sensitive detection of ClO^−^ in biological systems. This probe leverages structure-responsive optical changes and demonstrates a pronounced fluorescence turn-on response to ClO^−^. It exhibits good selectivity for ClO^−^ over other tested anions and ROS. However, it is important to note that the effects of (hypobromous acid [HOBr] and peroxynitrite (ONOO^−^) on the probe were not evaluated in this investigation. Upon the introduction of ClO^−^, the probe displayed a distinct response, with fluorescence intensity increasing rapidly and stabilizing within one minute.

With low cytotoxicity, the probe XY-01 was utilized for real-time imaging of ClO^−^ distribution in HCT-116 cells and colorectal cancer organoids. Notably, these PDOs have shown the ability to accurately replicate tissue functions and structures *in vivo*, which is essential for their reliability and relevance in preclinical studies [40, 41]. The extent to which they mimic the physiological and pathological characteristics of native human tissues underscores their significance in research [42, 43]. Consequently, the novel fluorescent probe XY-01 holds promise for detecting ClO^−^ levels in living organisms. Its long-term applicability and low cytotoxicity highlight its potential for live-cell and live-organoid imaging applications. Cytotoxicity assays conducted at 12 and 24 h demonstrated that cell viability remained above 95% at concentrations ≤ 200 µM for live-cell imaging and up to 400 µM for live-organoid imaging, confirming its favorable biocompatibility profile. The low-dose compatibility minimizes cellular disturbance in single-cell models, while high-dose tolerance ensures adequate tissue penetration and stable signal acquisition in three-dimensional organoid systems. These properties enable the probe to bridge the gap between traditional cellular imaging and advanced organoid-based studies that replicate *in vivo* microenvironments. Collectively, these attributes establish a solid foundation for the probe’s future application in long-term dynamic tracking of biological processes across cellular and organoid models.

However, the present study has limitations, including the lack of evidence confirming that the observed fluorescence arises from endogenously generated ClO^−^, as HCT-116 cells and PDOs were not characterized for MPO expression or activity. Furthermore, the absence of whole-animal validation leaves the performance of probe XY-01 in intact physiological or pathological environments untested. Long-term photostability assays were also not conducted in this study to further validate the probe’s performance.

## Conclusion

In this study, we successfully designed and synthesized a novel A-D-A type fluorescent probe, XY-01, for the highly selective and sensitive detection of ClO^−^ in biological systems. XY-01 features a thioformyl group (–C=S) that serves as both the reactive moiety and the core unit disrupting ground-state ICT. The results indicate that probe XY-01 can rapidly respond to ClO^−^ within biologically relevant concentration ranges, with fluorescence at 666 nm reaching a plateau within one minute, demonstrating excellent selectivity and low detection limits. Mechanistically, XY-01 reacts with ClO^−^ to form XY-01-OH, facilitating effective ClO^−^ quantification in biological matrices. Further investigations suggest that probes like XY-01 hold significant potential for imaging ClO^−^ in living cells and organoids. Under the experimental conditions employed in this study, XY-01 also exhibited adequate photostability to support endpoint imaging. In summary, we present a novel molecular fluorescent probe for the optical sensing and imaging of ClO^−^ within living cells and organoids, characterized by simple operation, low toxicity, appreciable sensitivity, high selectivity, and adaptability to physiological conditions. This offers strong technical support for early disease diagnosis and prevention in the biomedical field.

## Supplemental data

Supplemental data are available at the following link: https://www.bjbms.org/ojs/index.php/bjbms/article/view/13312/4083.

## Data Availability

Dataset available on request from the authors.
